# From “carbohydrate” to standardized Chinese terminology: historical evolution and implications for nutrition communication

**DOI:** 10.3389/fnut.2026.1827194

**Published:** 2026-04-21

**Authors:** Shu Zeng, Hui Zhang, Peng Miao

**Affiliations:** 1 College of Foreign Languages, University of Shanghai for Science and Technology, Shanghai, China; 2 School of Health Science and Engineering, University of Shanghai for Science and Technology, Shanghai, China

**Keywords:** carbohydrate, China, history, nutrition communication, terminology, translation

## Abstract

The Chinese translation of “carbohydrate” has long been a topic of considerable debate in chemistry, biomedicine, and nutrition-related disciplines. This issue is not merely linguistic. In Chinese-language contexts, inconsistency among carbohydrate-related expressions may create ambiguity in nutrition education and public understanding, and may introduce practical challenges for literature retrieval and interdisciplinary collaboration, especially in fields such as type 2 diabetes mellitus (T2DM), where distinctions among dietary carbohydrates, sugars, and glucose could be crucial. This article traces the historical evolution of the Chinese translation of “carbohydrate” to clarify its historical trajectory and scientific implications. Historical evidence demonstrates that the term “carbohydrate” did not appear in dictionaries or chemistry books published prior to 1900. However, at the turn of the 20th century, multiple translations emerged, most of which were influenced by the Japanese term “tansuikabutsu/炭水化物.” The earliest recorded Chinese translation appeared in *Huaxue Yuanliu Lun*. During the early Republic of China, “tanshui huawu/炭水化物” became the most commonly used term, which was later revised around 1920 with the addition of a semantic radical to the character “tan.” In 1932, the National Institute for Compilation and Translation introduced the term “tang/醣,” which gained popularity alongside “tanshui hua(he) wu.” However, “tang” was officially abolished in the mid-to-late 1950s and gradually phased out in subsequent decades. By 1980, “tanshui huahe wu/碳水化合物)” and “tang lei/糖类” were officially established as equivalent translations. Currently, “tang lei” is preferred in some disciplinary standards, although “tanshui huahe wu” remains widely used by convention. By reviewing this history, the present work highlights three key principles for addressing terminological ambiguity in nutrition communication. While this historical narrative is anchored in the Chinese context, the communication risks and mitigation strategies discussed might be relevant to other cross-lingual or cross-disciplinary setting, where everyday dietary language interfaces with technical biomedical terminology.

## 1 Introduction

The translation of scientific terminology plays a pivotal role in the global dissemination of scientific knowledge, facilitating effective communication and collaboration across linguistic and cultural boundaries. In recent years, increasing scholarly attention has been paid to examining the evolution of terminology as a means to trace conceptual development within cross-cultural contexts. Such studies have flourished across various disciplines, including the field of biomedical science, where the historical trajectory of disease nomenclature has been a prominent area of research (1).

Similarly, in the field of glycoscience, where the study of carbohydrates and glycoconjugates bridges biology, chemistry, medicine, and nutrition, standardized terminology is indispensable for ensuring clarity and accuracy in research. Conceptual-historical approaches to the transcultural “travel” of biomedical terms—illustrated by case-based investigations of how these terms, such as “diabetes mellitus” and “diabetes insipidus,” were translated, localized, and standardized in modern China (2)—also provide a useful lens for examining the Chinese reception of foundational biomedical and nutritional categories.

This article explores the historical evolution of “carbohydrate” in China by tracing its translations over time and analyzing their implications for scientific communication. Specifically, we aim to clarify how the historical trajectory of “carbohydrate” has been shaped by linguistic, institutional, and scientific developments, and to discuss how the evolving translations of this term have influenced it scientific and public understanding in modern Chinese contexts. Beyond documenting a regional terminology history, we use this traceable case to illustrate a transferable issue in global scientific exchange: when technical terms move across languages and cultures and between expert and public registers, lexical overlap may obscure conceptual boundaries, potentially shaping how nutrition messages are received and interpreted.

The translation of “carbohydrate” into Chinese has been a contentious issue in the field of chemistry. A key challenge lies in the literal translation of the term as “tanshui huahe wu,” literally “hydrates of carbon,” which misleadingly implies that carbohydrates are composed of carbon and water; this is a misconception regarding their chemical structure. While some carbohydrates conform to the general formula Cm(H_2_O)n, others, such as deoxyribose (C_5_H_10_O_4_), do not, despite sharing similar properties. In light of this, the Committee on Chemistry Nomenclature (China) provided the following explanation for “tanshui huahe wu” in the latest edition of *Chemical Nomenclature* issued in 2016: compounds primarily composed of carbon, hydrogen, and oxygen, which contain polyhydroxy aldehydes or ketones. Their molecular formula is generally expressed as Cm(H_2_O)n, although exceptions, such as deoxyribose and rhamnose (C_6_H_12_O_5_), exist. Therefore, compounds containing polyhydroxy aldehydes or ketones are currently referred to as “tang/糖” (3). This shift underscores the evolving understanding of “carbohydrates” and their chemical properties.

However, even within the standardized terminology issued by authoritative national bodies in China, inconsistencies persist regarding the Chinese term for “carbohydrate.” In “Approved Terms Database” issued by China National Committee for Terminology in Science and Technology, which can be accessed on Termonline (www.termonline.cn), different categories of scientific disciplines adopt different normative terms for “carbohydrate.” In the *Terminology of Food Science and Technology* (2020) and *Terminology of Animal Science* (2020), the approved term is “tanshui huahe wu,” whereas in the *Terminology of Biochemistry and Molecular Biology* (2024) and *Terminology of Endocrinology and Metabolic Diseases* (2025), the approved term is “tang lei/糖类,” with “tanshui huahe wu” listed as an alternative name. This discrepancy highlights that even within the official terminology of the Chinese language, there remains a lack of uniformity in the representation of “carbohydrate.”

The question of whether “carbohydrate” should be translated as “tanshui huahe wu” or “tang lei” is less a matter of linguistic translation than one of the original term’s nomenclature. In 1831, British chemist William Prout referred to a class of sugar-containing foods as “hydrates of carbon” (4). In 1844, German chemist Carl Schmidt introduced the term “Kohlenhydrate,” the German equivalent of “carbohydrate” (5). At the time, scientists observed that many commonly known compounds could be represented by the general formula Cm(H_2_O)n, leading to the conclusion that these substances were composed of carbon and water, thus coining the term “carbohydrate.”

Nevertheless, by 1893, W. E. Stone, a prominent expert in carbohydrate research, published an article in *Science*, specifically discussing the issue of the term “carbohydrate.” He argued that “we have reached, or already passed, a transition stage in the use of the term ‘carbohydrate,’” as its nomenclature and definition neither encompassed newly discovered related substances nor accurately described the chemical properties of such compounds (6). Stone’s critique resonates with the current discussions within Chinese academia regarding the translation of “carbohydrate.” Indeed, determining the appropriate Chinese translation for “carbohydrate” has been long and intricate, shaped by historical, linguistic, and scientific developments. This article divides the evolution of the term into five distinct stages, tracing its trajectory from its origins in Western chemistry to its eventual adoption and adaptation in Chinese scientific discourse.

## 2 Review methodology

To achieve the objectives, we adopted a thematic historical framework to trace the evolution of carbohydrate-related terminology in Chinese and to analyze its implications for contemporary scientific communication. This approach involved a comprehensive and systematic examination of historical materials, focusing on three main types of sources.

The first type comprises bilingual and multilingual dictionaries. These include dictionaries primarily from the late Qing and Republican periods, which provided early renderings of scientific terms, including “carbohydrate.” Such dictionaries were instrumental in shaping the initial introduction of western scientific concepts into the Chinese language and offer valuable insights into the linguistic strategies employed during this period.

The second type comprises translated works and chemistry textbooks. These encompass translations of western scientific texts and chemistry textbooks published in Chinese during the late 19th and early 20th centuries. They reflect the evolving understanding of carbohydrate-related concepts and their integration into Chinese scientific discourse, as well as the influence of Japanese translations during the same period.

The third type comprises normative documents on chemical nomenclature and terminology standardization. This category includes official and semi-official documents produced by academic institutions, particularly those related to the standardization of chemical nomenclature in the early-to-mid-20th century. These documents provide a critical perspective on the institutional efforts to unify and standardize scientific terminology in Chinese.

Through close reading and qualitative analysis of these materials, we examined and compared how the term “carbohydrate” was rendered, defined, and explained across different historical periods and source types. This involved inspecting the linguistic choices made in translating the term, the scientific contexts in which it was used, and the institutional factors influencing terminology standardization.

Unlike systematic reviews that rely on predefined search strategies, this article employed an exhaustive approach to analyze a curated collection of relevant historical documents. These materials were drawn from accessible archives, libraries, and published collections, and were selected based on their historical significance, relevance to the topic, and the level of detail they provided about carbohydrate-related terminology. While not exhaustive in the strict sense of covering every possible document, the selection aimed to include key milestones in the development of Chinese scientific terminology for carbohydrates.

By synthesizing insights from these source categories, we reconstructed a concise history of the Chinese translations for “carbohydrate” and highlighted their implications for contemporary nutrition communication. More broadly, this thematic historical framework may serve as a model for addressing similar issues in other cross-lingual or cross-disciplinary contexts, where inconsistent keyword choices and register shifts may complicate cross-study synthesis, hinder literature retrieval, and pose challenges for translating evidence into practice.

## 3 Review results

### 3.1 Before 1900: the absence of a Chinese translation for carbohydrate

A search of the Chinese-English Dictionary Database maintained by the Institute of Modern History, Academia Sinica, reveals that early English-Chinese dictionaries published before 1900 did not include the term “carbohydrate.” However, the term “hydrate” was found in the *English and Chinese Dictionary* (1866–1869), edited by German missionary Wilhelm Lobscheid, as well as in an expanded edition published in 1884 by Inoue Tetsujirō. In these dictionaries, “hydrate” was defined as “a compound formed by metal oxides and water” and translated into Chinese as “shui yang jin/水养金” (7).

Early prominent works on inorganic chemistry, such as *Huaxue Chujie* (Elementary Introduction to Chemistry, 1870) and *Huaxue Jianyuan* (Principles of Chemistry, 1872), did not mention carbohydrates. *Huaxue Jianyuan Xubian* (Supplement to Principles of Chemistry), translated by John Fryer and Xu Shou, is regarded as the first Chinese translation dedicated to organic chemistry. Published in the first year of the Guangxu reign (1875), this book was based on Charles Loudon Bloxam’s 1867-work *Inorganic and Organic, with Experiments and a Comparison of Equivalent and Molecular Formulae*. Although the original text included chapters entitled “Starch,” “Bread” and “The Sugars,” it did not classify these substances as carbohydrates or mention terms such as “carbohydrate” or “hydrate of carbon” (8). The contents of *Huaxue Jianyuan Xubian* include corresponding sections labeled “Chapter 7: Starch (小粉),” “Chapter 8: Bread (馒头),” and “Chapter 9: Sugars (糖类)” (9). It is worth noting that later editions of the original text, such as the 11th edition published in 1923, included a dedicated section on carbohydrates.

Other early chemical texts published during the 1870s and 1880s, including *Huaxue Fenyuan* (The Elements of Chemistry, 1872), *Gezhi Qimeng: Huaxue* (Enlightenment of Science: Chemistry, 1879), *Huaxue Kaozhi* (Chemical Examination of Substances, 1883), *Huaxue Qiusu* (Chemical Quantitative Analysis, 1883), and *Huaxue Cailiao Zhongxi Mingmu Biao* (A Comparative Table of Chinese and Western Chemical Materials, 1884), were primarily translations of works on inorganic or analytical chemistry. While some of these texts mentioned substances such as sugars and starch, they did not classify them under the category of carbohydrates.

### 3.2 1900–1911: the proliferation of Chinese translations, influenced by the Japanese term “tansuikabutsu/炭水化物”

In the early 20th century, various translations of the term “carbohydrate” appeared in chemistry texts, with corresponding expressions traceable in the original works. The earliest example can be found in the translated biochemical text *Huaxue Yuanliu Lun* (On the Origins and Development of Chemistry), published in the 26th year of the Guangxu reign (1900). This work was translated by Wang Ruran and based on *Chemistry, as Exemplifying the Wisdom and Beneficence of God*, an 1844-work by British chemist George Fownes. The “Biochemistry” section in Volume 1 of *Huaxue Yuanliu Lun* mentions that sugars could be referred to as “tan yu shui he,” literally meaning “carbon and water combined,” “tan yu qing yang,” literally meaning “carbon, hydrogen, and oxygen” or “tan qing yang he zhi,” literally meaning “a compound of carbon, hydrogen, and oxygen,” but not as “tan yu shui,” literally meaning “carbon and water.” The term “hydrate of carbon” in the original text corresponds to the translation “tan yu shui he” in the Chinese version (10, 11).

Another early work involving the term “carbohydrate” was the *Zuixin Zhongxue Jiaoke Shu Huaxue* (The Latest Chemistry Textbook for Middle Schools), published in the 29th year of the Guangxu reign (1903). This book was translated by the Zhongxi Publishing House from *Popular Chemistry*, an 1897-work by J. Dorman Steele. In this book, the chapter titled “han tan yang qing zhi zhi,” literally meaning “substances containing carbon, oxygen, and hydrogen,” corresponds directly to the chapter titled “The Carbohydrates” in *Popular Chemistry* (12, 13). The translation “han tan yang qing zhi zhi/含炭养轻之质” might have been influenced by *Huaxue Cailiao Zhongxi Mingmu Biao*, which translated “hydrates” as “han qing yang zhi zhi/含轻养之质,” literally meaning “substances containing hydrogen and oxygen” (14). The compiler of this text noted that the names of organic substances were often adopted from the translations of John Fryer.

After the Sino-Japanese War of 1894–1895, a wave of “Eastern learning” swept through China, leading to the translation of many chemistry textbooks from Japanese sources. The Japanese term for “carbohydrate,” “tansuikabutsu,” was often retained or slightly modified when adopted into Chinese due to the linguistic similarities between the two languages. For instance, *Zuijin Putong Huaxue Jiaokeshu* (The Latest General Chemistry Textbook), translated by Changsha Sanyi Society from the work of Japanese scholar Kameoka Tokuhira in 1904, used both “tan shui hua wu/炭水化物” and “han shui tan su/含水炭素” (literally meaning “carbon containing water”) (15). Similarly, *Zuixin Huaxue Jiaoke Shu* (The Latest Chemistry Textbook), translated by Wang Jilie from the work of Ōkō Yūkichi in 1906, adopted the translation “tan zhi shui hua zhi / 炭之水化质,” literally meaning “substance of carbon’s hydration” (16).

In *Huaxue Xin Jiaoke Shu* (New Chemistry Textbook, 1905) translated by Du Yaqian, and *Zuixin Huaxue Jiaoke Shu* (The Latest Chemistry Textbook, 1907) translated by He Yushi, both based on the works of Yoshida Hikoroku, different translations of “carbohydrate” were employed. In Section 96 of Chapter 42, Du translated it as “tan shui he zhi/炭水合质,” literally meaning substance of carbon and water (17), while He used the term “tan shui huahe wu” (18).

In addition to chemistry textbooks, the term “carbohydrate” was also included in *Deutsch-Englisch-Chinesische Wissenschaftliche Terminologie* (German-English-Chinese Scientific Dictionary), compiled by German sinologist Richard Wilhelm in 1911, where it was translated as “tan shui hua wu” (19). Notably, *An English and Chinese standard dictionary* compiled by Yen Wei-ching in 1908, added the translation “shui hua wu” under the entry for “hydrate,” which might have contributed to the gradual standardization of the term “tan shui hua wu” (20).

From the above, it is evident that during the final years of the Qing Dynasty, there was no consistent translation for “carbohydrate.” Most translations were similar in form to the Japanese term “tansuikabutsu.” As Du Yaqian noted in his preface, the naming of inorganic substances was “no easy task, with some names following old translations and others being newly coined, resulting in a lack of systematic order (17)”.

### 3.3 1912–1931: the prevalence of “tan shui hua wu” and its revision

At the beginning of the Republic of China, there was no dedicated institution to oversee the translation of scientific terminology, resulting in inconsistent and disorganized terminology. In 1918, the Ministry of Education convened a meeting of secondary school principals in Nanjing. The conference submitted a proposal to the Ministry of Education to establish an institution for the unification of scientific terminology. Consequently, the “General Committee for Medical Terminology” was expanded and renamed the “General Committee for Scientific Terminology” in December of the same year.

Between 1912 and 1920, chemistry textbooks and reference works frequently translated “carbohydrate” as “tanshui hua(he) wu.” For example, Wang Jianshan’s *Minguo Xin Jiaoke Shu Huaxue* (The Republic’s New Chemistry Textbook, 1913) classified sugars and starches as “tanshui hua(he) wu” (21). Similarly, Yun Fuseng’s *Xiangzhu Yinghan Huaxue Cihui* (Annotated English-Chinese Chemical Vocabulary, 1920) included the entry for “carbohydrate,” translating it as “tan shui hua wu” and noting that some carbohydrates did not conform to the general formula Cm(H_2_O)n (22).

In 1915, the Ministry of Education issued the *Wuji Huaxue Mingming Cao’an* (Draft Nomenclature for Inorganic Chemistry), which proposed standardizing the radicals used in the names of chemical elements. As part of this reform, the characters for the elements “carbon” (炭, pronounced as “tan”), “hydrogen” (轻, pronounced as “qing”), and “oxygen” (养, pronounced as “yang”) were revised to “tan/碳,” “qing/氢,” and “yang/氧,” respectively. Between 1921 and 1932, most chemistry textbooks adopted these standardized radicals in their terminology (23). Consequently, textbooks published after 1920 often translated “carbohydrate” as “tan shui hua wu/碳水化物.” For instance, the 19th edition of Wang Jianshan’s *Minguo Xin Jiaoke Shu Huaxue* (New Republican Textbook: Chemistry), published in 1922, revised the term “tanshui hua(he) wu/炭水化(合)物” to “tan shui wu/碳水物.” Similarly, Zheng Zhenwen and Zheng Zunfa’s *Xinzhuan Chuji Zhongxue Jiaoke Shu Huaxue* (Newly Compiled Chemistry Textbook for Junior Middle Schools, 1928) also adopted the term “tan shui hua wu/碳水化物” as the translation for “carbohydrate” (24).

### 3.4 1932–1979: the creation, abandonment, and decline of the term “tang/醣”

In 1932, the establishment of the National Compilation and Translation Bureau marked a new era in the standardization of chemical nomenclature in China. The following year, the bureau published *Huaxue Mingming Yuanze* (Principles of Chemical Nomenclature), which stipulated: “Compounds that can be represented by Cm(H_2_O)n are collectively referred to as tang/醣 (carbohydrates) (25).” There were two possible reasons for the creation of the character “tang.” First, it was intended to serve as a generic term to distinguish it from “tang/糖”, the equivalent to “sugar”, as in the case of monosaccharides and glucose, which belong to this category. Second, the translation of “carbohydrate” had not yet been unified, and the term “tan shui (hua) wu/碳水 (化)物” was considered insufficiently precise.

The 1945 edition of *Principles of Chemical Nomenclature* revised the name and definition of the term. It stated: “Compounds containing aldehyde-alcohol or ketone-alcohol groups, or those that can hydrolyze into such compounds, are collectively referred to as tang (醣). Their molecular formula can often be expressed as Cm(H_2_O)n, and they are therefore also called tanshui huahe wu (carbohydrates)” (26). At this point, the translation of “carbohydrate” was officially established as either “tang (lei)/醣 (类)” or “tanshui huahe wu/碳水化合物.”

On June 13th, 1955, the Biochemical Terminology Joint Conference, organized by the Compilation and Translation Committee, Chinese Academy of Sciences in Beijing, decided to abolish the character “tang/醣,” a decision that was formalized through official notices issued in the late 1950s. The meeting concluded that “tang/醣” and “tang/糖” were homophones and that there was no need to create this new character (27). As a result, the former was abandoned, and “carbohydrate” or “saccharide” was officially designated as “tanshui huahe wu/碳水化合物,” while “tang lei/糖类” was used to refer to the sugar subset of carbohydrates.

### 3.5 1980–present: the official establishment of the terms “tang lei (huahe wu)/糖类(化合物)” and “tanshui huahe wu/碳水化合物”

The 1980 edition of *Principles of Organic Chemistry Nomenclature* classified structures containing polyhydroxy aldehydes or ketones under the general term “tang/糖,” equating saccharide compounds with carbohydrates (28). This indicates that “tang lei/糖类” was no longer limited to the sugar subset within carbohydrates. In fact, as early as the 1960s, many chemistry textbooks had explicitly pointed out that “tang/糖” was a synonym for “tanshui huahe wu,” as seen in widely used textbooks such as *Youji Huaxue Jichu* (Fundamentals of Organic Chemistry, 1965) authored by Xing Qiyi (29). At this point, the terms “tang lei” and “tanshui huahe wu” were officially established.

The 2017 edition of *Principles of Organic Compound Nomenclature* directly replaced “tanshui huahe wu” with “tang” as the chapter title, providing a more detailed explanation of the chemical substances it encompasses. It also included the following note: “The term “tang/糖” was previously known as “tang/醣”, which was abolished after 1980. However, in recent years, there have been suggestions to reinstate the use of “tang/醣” as an academic term to denote a category corresponding to “carbohydrate” and “saccharide” (30). The phrase “abolished after 1980” reflects the original wording in this 2017 document; historically, the abolishment decision was made in 1955 and formalized through official notices in the late 1950s, although the term continued to appear in some later publications.

**Figure 1:**
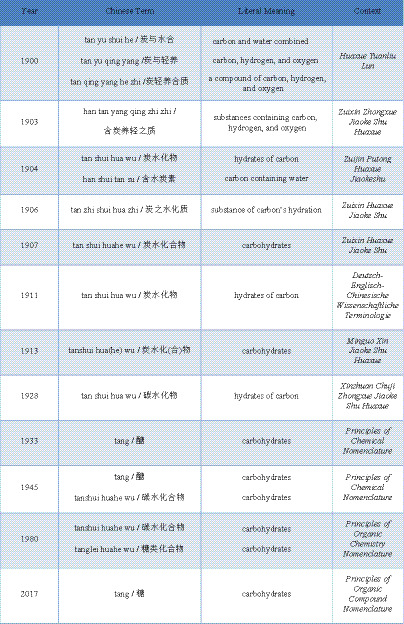
Historical trajectory of “carbohydrate” translations in Chinese.

Today, “tang lei” is widely used in many contemporary Chinese-language contexts, whereas “tanshui huahe wu” remains common in others, partly due to convention and disciplinary preference. Figure 1 provides an overview of the historical trajectory of “carbohydrate” translations in Chinese.

## 4 Discussion and conclusion

Tracing the evolution of “carbohydrate” in Chinese helps clarify both the distinctions and the continuities among competing translations, and can illuminate where terminological misuse or conceptual confusion is likely to arise. More broadly, this century-long history offers insights into how scientific terminology is standardized and harmonized in practice. For instance, the revision from “tan shui hua wu/炭水化物” to “tan shui hua wu/碳水化物” highlights the expressive characteristics of the Chinese language, aligning with broader language-modernization efforts. By contrast, later shift from “tanshui huahe wu/碳水化合物” to “tang lei huahe wu/糖类化合物” reflects attempts to reconcile established usage with a more conceptually adequate understanding of the category. These shifts illustrate the dynamics of linguistic, institutional, and scientific developments, which has shaped the standardization of carbohydrate-related terminology in Chinese.

This history matters for nutrition communication because “carbohydrate” is a foundational category used to describe dietary composition, energy metabolism, and diet-related strategies. Yet, in Chinese-language communication, “tang/糖” is frequently used in an undifferentiated way that overlaps with “sugar” in everyday speech. As a consequence, public cognition may shift toward an exclusive focus on “sweet foods” and away from a fuller understanding of carbohydrate intake (including starch-rich staples), which may reduce the clarity and consistency of dietary guidance. In this sense, terminology can be viewed as part of the infrastructure of effective nutrition communication, as it may influence what lay audiences think dietary advice refers to, and how they interpret recommendations.

The implications of this history extend to metabolic disease contexts, including T2DM. In T2DM-related education and counseling, common advice may include “controlling carbohydrates,” “reducing sugar,” and “monitoring blood glucose.” These instructions operate at different conceptual levels: “carbohydrates” (“tang lei” as a possible translation) refer to nutrient intake; “sugar” (“tang” in Chinese) usually refers to specific dietary components, or sweeteners in people’s everyday diets; “blood glucose” (“xue tang” in Chinese) refers to a measurable metabolic indicator. If Chinese terms for these concepts, all containing “tang,” are used loosely without explicit differentiation, communication may become ambiguous at the point of counseling and education. In research contexts, similar ambiguity may complicate literature retrieval and pose challenges for interdisciplinary cooperation. A recent public-facing nutrition interview in a national food-industry newspaper illustrates how these concepts are often presented together in Chinese, explicitly cautioning against the slogan “duan tang jue tan/断糖绝碳” (literally meaning “cutting sugar and eliminating carbohydrates”) while discussing carbohydrate intake, added sugar limits, and glycemic management in the same narrative (31). Such materials do not demonstrate effects on study outcomes, but they provide a concrete example of why terminological clarification might be repeatedly needed in T2DM-related education and counseling.

At the same time, the contemporary coexistence of “tanshui huahe wu” and “tang lei” does not necessarily imply that one must be abolished in favor of the other. The historical record suggests that different terms gained authority in different contexts for different reasons. Therefore, a practical approach is to promote context-sensitive clarity rather than to impose an artificial uniformity. Based on the historical trajectory reviewed in this article, three principles may be proposed. First, “tanshui huahe wu” might be more suitable and useful for nutrition education and public communication, given its broad recognition and alignment with conventional nutrition discourse; second, “tang lei” might be preferable in some research contexts, particularly biochemistry and glycoscience, where chemical classification and biochemical function are foregrounded. Third, indiscriminate use of “tang” as an umbrella term should be avoided to minimize conceptual slippage between dietary intake, metabolic regulation, and measurement. By applying these principles, researchers, educators, and policymakers can work toward reducing ambiguity and improving clarity of nutrition communication in Chinese.

## 5 Final remarks

This article has traced the historical trajectory of “carbohydrate” terminology in Chinese, from its initial introduction in western chemistry to its adoption and adaptation in Chinese scientific discourse, and has demonstrated how historical developments in terminology may shape contemporary scientific communication and nutrition education. The implications of this study extend beyond the specific case of “carbohydrate” in Chinese: as technical terms move across languages and cultures, they often encounter challenges such as lexical overlaps, register shifts, and conceptual ambiguities. This case highlights the importance of context-sensitive approaches to terminology standardization, which reconcile historical usage with scientific accuracy and practical applicability.

While this study does not empirically evaluate the impact of terminological choices on educational or clinical outcomes, it may provide clues for future research to systematically assess the role of terminology in shaping public health communication and interdisciplinary collaboration. Ultimately, this article underscores the critical role of terminology as a bridge between language, science, and society, and highlights the need for ongoing efforts to clarify scientific language in an increasingly interconnected world.
